# Shell Wound in the Forehead

**Published:** 1867-12

**Authors:** A. A. Dunn


### SHELL WOUND IN THE FOREHEAD.

By A. A. Dunn, M.D.

It has been suggested by some medical gentlemen that a condensed statement of the case with the above caption, read before the Chicago Medical Society in April last, would not be uninteresting to the profession, and, as I have seen a very brief synopsis of that report in the *Chicago Medical Journal*, and not altogether correct, I am induced to submit the following for publication in the same journal, as it gives a better idea of the facts of the case than was given by the synopsis referred to:

The wound was received at the battle of Franklin, Tenn., 30th Nov., 1864. The missile was a fragment of shell which struck the forehead some two inches above the eye, and penetrated the frontal sinus. Complete insensibility was produced at the time, and was followed by periods of delirium, principally upon waking out of sleep, for four weeks. The delirium, however, was not so complete as to prevent the subject remembering what transpired during the aberration. No paralysis followed, save over the scalp in the region of the wound; which region was, in a few days, assailed by the most intolerable itching, radiating from the wound. The propensity to rub, scratch, and dig at the part affected thus, was irresistible, but, for many days, even weeks, rubbing a board would have answered equally as good a purpose toward allaying the itching.

Three days after the injury was received, several spicula of bone were removed from the wound. It has remained open, except for brief periods, ever since; but at no time was any loose bone discovered in the wound, or in the discharges, till the 19th day of March last, just two years, three months, and nineteen days from date of injury, when a few small fragments of carious bone were discharged through the nostril. The wound was several times probed with the view to find carious bone, but the impulse of the probe upon the bone within and without the sinus, at no time revealed any carious surface. Since the expulsion of those fragments, the wound has remained open nearly all the time, the discharge being much less than formerly, purulent, and inodorous, generally. Not so much can be said for what passed through the nostril, which was small in quantity, but occasionally quite fetid.

Within two weeks after the fracture, neuralgia, violent and intolerable, assailed the head and face. Sometimes the attacks were sudden and brief, at other, gradual and prolonged, these latter invariably producing torpor of greater or less duration, and out of which the patient issued, at times, at once, and completely refreshed, and, then again, depressed and languid in body and mind. These paroxysms continue to recur, but with greatly decreased violence and frequency.

For six months the head suffered from a sense of constriction; 'twas as though a band of iron was around the head alternately tightened and relaxed; and during that time, it was impossible for the subject to tell, without putting his hand to his head, whether or not his hat was on. To this day, that condition of things recurs—at long intervals, however.

Three weeks after date of injury, the patient returned alone to his home in Illinois. But for a week after arriving there, it was a question if a grave error had not been committed by leaving the hospital. The process of recovery was, however, soon reëstablished, and in February, during very cold weather, he rejoined his regiment, and was discharged with it, the June following, in North Carolina.

Upon the accession of the neuralgia, morphia, in large doses, was resorted to for relief from the terrible suffering. Apprehension, in the mind of the patient, as to the effect of the opiate upon the bowels, and as a habit, induced him to resort to hot whisky punches, within a week after commencing the morphia, and with a tolerably happy effect, the latter being resorted to only occasionally, and not at all within the last year.

No general treatment was pursued. Not a dose of medicine of any kind was taken, save the articles mentioned. The local treatment was water dressing. Oakum, for three months, was placed on the wound to absorb the discharges, and afterwards, lint was used. For more than a year past, no dressing has been used, a handkerchief being carried for the special purpose of looking after the discharge, which, of course, has not been much. During the winter of 1865 and 1866, the fistulous opening was deeply cauterized with a stick of the solid nitrate of silver, producing a paroxysm of neuralgia, which rendered the patient nearly frantic, and led to the taking of enormous doses of sul. mor. for relief. Subsequently, the same material, in solution, and of variable strength, at no time exceeding 10 grs. of the salt to the oz. of water, was injected a few times, ralways inducing ascally neuralgic paroxysms, and was not persisted in.

The suggestion to cut down upon the bone and chisel out any carious bone that might be found, was several times made, but never entertained by the patient.

After the first few weeks from the injury, the general health of the patient, baring the neuralgia, has been absolutely good.

The points of interest in this case are:—

The rapid recovery under circumstances where no particular effort was made to avoid exposure to temperature and other conditions of weather;

The perfect impunity with which morphia and alcoholic stimulants were used so soon after the head was wounded;

And, the soundness, or unsoundness, of the let alone process pursued in the case.

On the second point mentioned, I may be permitted to say, the effect of the alcohol upon the cerebral mass, would seem to verify the theory of Dr. N. S. Davis, that alcoholic stimulants, are *not* stimulants, but are sedative and anæsthetic in their action upon the nervous system.

In addition to the phenomena mentioned, it may be well to state, that, the concussion had the effect to quicken and intensify a naturally irascible temper, it is to be feared, permanently.

